# Influence of Lithium Triflate Salt Concentration on Structural, Thermal, Electrochemical, and Ionic Conductivity Properties of Cassava Starch Solid Biopolymer Electrolytes

**DOI:** 10.3390/ijms25158450

**Published:** 2024-08-02

**Authors:** Alvaro A. Arrieta, Oriana Palma Calabokis, Carlos Vanegas

**Affiliations:** 1 Department of Biology and Chemistry, Universidad de Sucre, Sincelejo 700001, Colombia; 2 Faculty of Engineering and Basic Sciences, Fundación Universitaria Los Libertadores, Bogotá 111221, Colombia; opalmac@libertadores.edu.co

**Keywords:** solid biopolymer electrolytes, cassava, starch, lithium triflate

## Abstract

Cassava starch solid biopolymer electrolyte (SBPE) films were prepared by a thermochemical method with different concentrations of lithium triflate (LiTFT) as a dopant salt. The process began with dispersing cassava starch in water, followed by heating to facilitate gelatinization; subsequently, plasticizers and LiTFT were added at differing concentrations. The infrared spectroscopy analysis (FTIR-ATR) showed variations in the wavenumber of some characteristic bands of starch, thus evidencing the interaction between the LiTFT salt and biopolymeric matrix. The short-range crystallinity index, determined by the ratio of COH to COC bands, exhibited the highest crystallinity in the salt-free SBPEs and the lowest in the SBPEs with a concentration ratio (Xm) of 0.17. The thermogravimetric analysis demonstrated that the salt addition increased the dehydration process temperature by 5 °C. Additionally, the thermal decomposition processes were shown at lower temperatures after the addition of the LiTFT salt into the SBPEs. The differential scanning calorimetry showed that the addition of the salt affected the endothermic process related to the degradation of the packing of the starch molecules, which occurred at 70 °C in the salt-free SBPEs and at lower temperatures (2 or 3 °C less) in the films that contained the LiTFT salt at different concentrations. The cyclic voltammetry analysis of the SBPE films identified the redox processes of the glucose units in all the samples, with observed differences in peak potentials (Ep) and peak currents (Ip) across various salt concentrations. Electrochemical impedance spectroscopy was used to establish the equivalent circuit model Rf–(Cdl/(Rct–(CPE/Rre))) and determine the electrochemical parameters, revealing a higher conduction value of 2.72 × 10^−3^ S cm^−1^ for the SBPEs with Xm = 17 and a lower conduction of 5.80 × 10^−4^ S cm^−1^ in the salt-free SBPEs. It was concluded that the concentration of LiTFT salt in the cassava starch SBPE films influences their morphology and slightly reduces their thermal stability. Furthermore, the electrochemical behavior is affected in terms of variations in the redox potentials of the glucose units of the biopolymer and in their ionic conductivity.

## 1. Introduction

Since Peter Wright’s pioneering discovery in 1975 of the conductivity of poly(ethylene oxide) (PEO) with sodium ions (Na+), solid polymeric electrolytes (SPEs) have emerged as a compelling alternative to liquid electrolytes, offering several notable advantages [1,2]. For example, one notable advantage is their enhanced safety; by eliminating the risks of leakage and evaporation inherent in liquid electrolytes, SPEs offer a more reliable and secure option. Furthermore, they demonstrate improved mechanical strength and thermal stability, ensuring long-term performance and reducing the need for complex packaging. Moreover, another key benefit is their higher ion conductivity, which facilitates efficient ion transport within the material, thereby enhancing the performance of energy storage and conversion devices. As a result, these advantages have positioned SPEs as a promising option for various applications. Since Michel Armand proposed them for use in lithium batteries [3], they have been included in other devices such as fuel cells, supercapacitors [4], sensors [5], and accumulators [6], among others.

On the other hand, solid electrolytes composed of biopolymers, known as solid biopolymer electrolytes (SBPEs), provide a significant benefit regarding environmental care. Specifically, biopolymers derived from renewable sources such as plant materials or microorganisms are biodegradable and have a lower environmental impact compared to the conventional petroleum-based polymers. Therefore, this makes SBPEs a more sustainable option for energy conversion and storage devices. Additionally, another advantage is their biological compatibility; SBPEs can be biocompatible and can be used in implantable medical devices, such as biosensors or drug delivery systems. For instance, the development of a biocompatible magnesium–air battery created with an SBPE of chitosan and choline nitrate has been reported [7], and a Zn–alginate polymer electrolyte was used to create a battery using an electro-crosslinking strategy [8]. Moreover, SBPEs exhibit excellent mechanical properties, such as flexibility and stretchability. This flexibility enables the fabrication of solid-state devices with complex shapes or conformal designs, which can be advantageous in wearable electronics or flexible energy storage systems. Consequently, SBPEs have been designed using biopolymers of starch [9,10], cellulose [11,12], chitosan [7,13], alginate [8,14], and peptins [15,16].

Due to its properties and natural abundance, starch stands out as a material of great interest in the development of solid biopolymer electrolytes (SBPE). Starch is a complex carbohydrate and one of the most abundant polysaccharides in nature. Its chemical structure consists of two distinct components: amylose and amylopectin. Amylose is a mainly linear chain of glucose molecules linked by alpha−1,4 glycosidic bonds, while amylopectin is a branched molecule with additional alpha−1,6 glycosidic bonds creating branch points [17]. The proportion of amylose and amylopectin can vary among different sources of starch. Starch is commonly found in plants like corn, potatoes, wheat, rice, and cassava, making it a readily available and renewable resource. Among these, cassava starch is a biopolymer with great potential for the production of SBPEs [9,18,19]. In particular, it can be an excellent alternative to synthetic polymers due to its abundance, low cost, and biodegradability. Moreover, the starch matrix provides a stable and interconnected network for ion transport, while the added salts enhance the overall ionic conductivity. Thus, this combination enables the development of SBPEs with desirable properties, such as high ionic conductivity, good mechanical strength, and electrochemical activity [18,20,21,22,23]. Consequently, cassava starch SBPEs have been used in devices such as electrochemical accumulators [24], artificial muscle [25], and supercapacitors [26].

Various formulations and synthesis conditions have been used in the preparation of starch SBPEs, revealing that the synthesis parameters and materials significantly influence the properties of the resulting biopolymers. For example, the impact of the glycerol concentration used in the synthesis of potato starch SBPEs on their electrical properties has been reported [27]. Similarly, the sequence in which the synthesis components are added has been found to affect the optical, mechanical, and electrical characteristics of corn starch SBPEs [28]. Additionally, different types of monovalent and divalent salts are commonly used to elaborate SBPEs. In particular, the cations of the dissociated salts coordinate with the oxygen atoms of the biopolymeric matrices to form biopolymer–salt complexes [29]. Different combinations of corn starch and lithium perchlorate (LiClO_4_) were mixed together in varying proportions, ranging from 10% to 50% by weight, in order to prepare SBPEs via the solution casting technique [29]. The best conductivity, measuring 1.28 × 10^−4^ S cm^−1^, was attained when the ratio was 40% LiClO_4_ and 60% corn starch, at a temperature of 353 K [29].

In the literature, there are a few studies regarding cassava starch-based SBPEs [23,30,31,32,33,34,35]. One study demonstrated that increasing the temperature (up to 100 °C) and glycerol concentration in glycerol/cassava starch SBPEs improved the electrical conductivity due to thermally activated DC-conduction and the coordination of glycerol’s –OH groups with starch chains [30]. Similarly, the addition of ammonium nitrate (NH_4_NO_3_) in SBPEs based on tapioca starch/polyethylene oxide [31] and cassava starch/chitosan [32] enhanced the conductivity by increasing the ionic mobility and the number of free ions. Another study used LiBF4 as a dopant salt, chitosan as a biopolymeric filler, and cassava starch as the polymer matrix, finding that increasing the LiBF_4_ concentration boosted the electrical conductivity, possibly due to chitosan’s properties [33].

More recently, the effects of different lithium salts (LiCl, Li_2_SO_4_, and CF_3_LiSO_3_) were investigated regarding the ionic conductivity, thermal behavior, and structural behavior of cassava starch-based SBPEs, revealing that CF_3_LiSO_3_-containing SBPEs exhibited higher molecular ordering, greater thermal stability, and lower redox potentials compared to those with LiCl and Li_2_SO_4_ [34]. Additionally, the studies concluded that incorporating cassava starch with synthetic polymers improved the properties of the solid polymer electrolytes, making them suitable for energy storage devices. This highlights the potential for research on cassava starch-based SBPE since it has not been well-established as to how the lithium salt concentration influences the properties of biopolymeric solid electrolytes. Therefore, the main objective of this work is to determine the effect of the lithium triflate salt concentration on the structural, thermal, electrochemical, and ionic conductivity properties of cassava starch SBPEs.

## 2. Results

### 2.1. FTIR Spectroscopy of Cassava Starch SBPE Films

**Figure 1:**
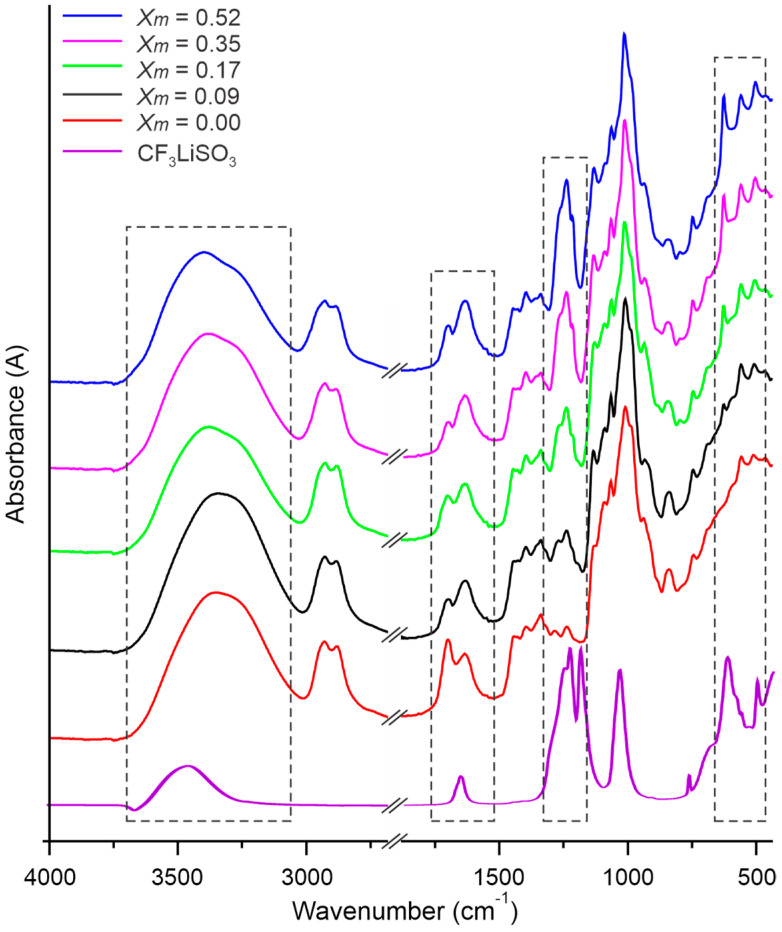
Infrared spectra of cassava starch salt-free SBPE films, with different LiTFT salt concentrations and pure LiTFT salt.

In order to analyze the effect of the LiTFT concentration on the structure and chemical composition of SBPEs, FTIR measurements were carried out. The infrared spectra of pure LiTFT and cassava starch-based polymer electrolytes (SBPEs), both without salt and with various LiTFT salt concentrations (Xm = moles of salt/moles of starch), are presented in Figure 1. Predominantly, the spectra of the SBPEs exhibit the characteristic bands of starch in all the samples. In the spectra, it can be observed that the presence of the LiTFT salt in the SBPEs generates a shift in the band corresponding to the OH group present in the area of 3100–3700 ${cm}^{-1}$ toward the position of the characteristic band of LiTFT, which is located at 3486 ${cm}^{-1}$. The presence of the OH band in the spectrum of the LiTFT salt may be due to the absorption of water during handling since it is a highly hygroscopic compound. As a result, the band attributed to OH vibrations in the salt-free SBPEs, originally at 3364 ${cm}^{-1}$, shifts to 3371, 3377, 3385, and 3399 ${cm}^{-1}$ in the salt-containing SBPEs, correlating to LiTFT concentrations of 0.09, 0.17, 0.35, and 0.52, respectively. Thus, with an increase in the LiTFT concentration within the SBPEs, the vibrations of the OH group are observed to shift to higher wavenumber values. This variation can be caused by the interaction of the ${Li}^{+}\leftarrow OH$ complex between the lithic ion from the LiTFT and the OH groups of the water present in the polymer matrix and also with the hydroxyl groups of the monomeric glucose units in the starch chains.

On the other hand, the band corresponding to C-H stretching present at 2926 and 2878 ${cm}^{-1}$ in the salt-free SBPE remained unchanged at 2927 and 2878 ${cm}^{-1}$, respectively, across all the SBPE samples containing lithium salt. The relative intensities of the absorbances for the bands at 1713 and 1649 ${cm}^{-1}$ assigned to C = O stretching vibration and O-H (water) bending vibration, respectively, are affected by the addition of the LiTFT salt. In the spectra of salt-free SBPEs, the band at 1713 ${cm}^{-1}$ appears to be more intense compared to the band at 1649 ${cm}^{-1}$. In the SBPEs with salt, this vibration is located at 1648 ${cm}^{-1}$ and the characteristic band of pure LiTFT at 1647 ${cm}^{-1}$. The presence of salt in the SBPEs enhances the absorbance of this band, making it more prominent than the band at 1713 ${cm}^{-1}$. This change is attributed to the overlap and subsequent increase in absorbance of the band at this region, which also includes the OH bending modes for the sulfonic group occurring at 1647 ${cm}^{-1}$. This overlap, intensified by the increased salt concentration, results in the marked amplification of the band’s absorbance.

Some of the LiTFT bands are evident in the spectra of the SBPEs with salt. Notably, the bands within the 1292–1248 and at 1229 ${cm}^{-1}$ ranges, corresponding to the asymmetric stretching mode of SO_3_ and the symmetric stretching mode of CF_3_ vibrations, are distinctly visible in the spectra of the SBPEs formulated with lithium salts. Additionally, their absorbances intensify in direct proportion to the increasing concentrations of the salts. In this spectral region, the presence of starch bands at 1247 and 1202 ${cm}^{-1}$, associated with CH_2_OH and COH vibrations, respectively, can be observed. These bands appear displaced and overlapping in the SBPEs with salt, presenting values of 1250 and 1219 ${cm}^{-1}$. Table 1 provides a comprehensive overview of the vibration bands identified in the spectra of pure LiTFT, and SBPEs both without salt and with varying salt concentrations.

The bands corresponding to the asymmetric stretching mode of CF_3_ at 1182 ${cm}^{-1}$ and the symmetric stretching mode of SO_3_ at 1039 ${cm}^{-1}\;$vibrations are indiscernible in the spectra of the SBPEs with salt due to the overlapping of the starch bands within the same spectral region. The characteristic bands of starch in this region are also observed at 1146 ${cm}^{-1}$ due to CO antisymmetric bridge stretching; 1103 ${cm}^{-1}$ due to the COH antisymmetric stretching in the plane ring; 1077 due to C-OH bending; and 1018 ${cm}^{-1}$ assigned to the COC ring vibration of the carbohydrates. The band at 1018 ${cm}^{-1}$ serves as an indicator of complexation between the biopolymer and the salt. Upon the addition of the LiTFT salt to the SBPE preparation mixture, it dissociates into ${{CF}_{3}{SO}_{3}}^{-}$ anions and ${Li}^{+}$ cations. The ${Li}^{+}$ cation interacts with the O atom of the COC groups within the biopolymeric matrix, forming salt–biopolymer complexes. This interaction is evidenced by a shift in the COC ring vibration of the carbohydrates from 1018 to 1021 ${cm}^{-1}$ following the salt addition. This shows an interaction between the salt and the biopolymer in a coordination bond, indicating a complexation.

The band at 627 ${cm}^{-1}$ assigned to the symmetric bending mode of the SO_3_ present in the spectrum of pure LiTFT is shown in the spectra of the SBPEs with salt at 638 ${cm}^{-1}$, while the vibrations of the asymmetric bending mode of CF_3_ at 574 ${cm}^{-1}$ and asymmetric bending mode of SO_3_ at 512 ${cm}^{-1}$ are not clearly observed in the spectra of the SBPEs with LiTFT because they overlap with the bands of the pyranose ring vibrations of cassava starch that are present at 570 and 522 ${cm}^{-1}$.

**Figure 2:**
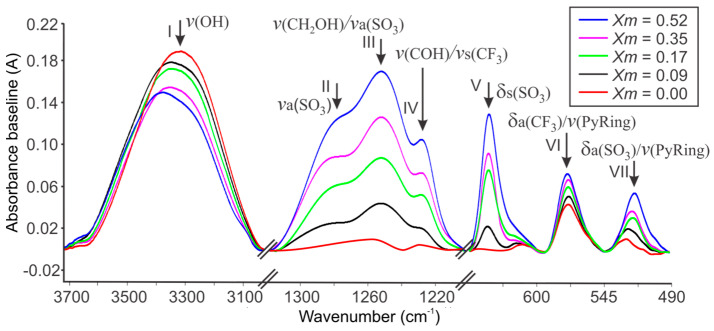
Magnification of segments of FTIR-ATR spectra of the SBPEs with different LiTFT salt concentrations.

**Figure 3:**
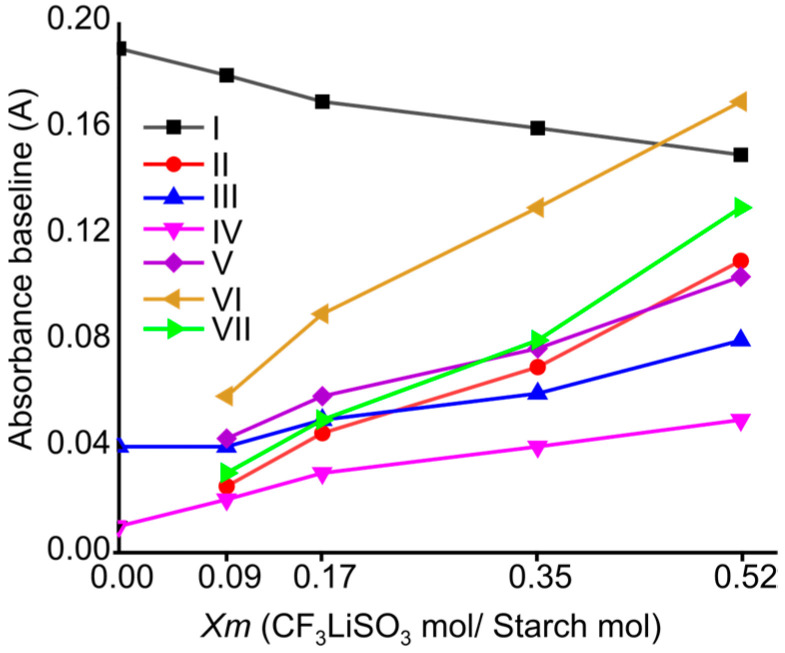
Normalized absorbance of the bands presented in Figure 2 versus LiTFT salt concentration in the SBPEs.

The FTIR spectra of the SBPEs with different concentrations enabled establishing the effect of the amount of salt on some of the bands related to the structure of the biopolymers. Figure 2 shows the expanded regions of the spectra, highlighting the bands where significant changes due to the addition of LiTFT salt at varying concentrations are observed. These include the band corresponding to the O–H stretching vibration (I); the asymmetric stretching mode of SO_3_ (II); the CH_2_OH-related modes/asymmetric stretching mode of SO_3_ (III); the bands assigned to the COH deformation/symmetric stretching mode of CF_3_ (IV); the symmetric bending mode of SO_3_ (V); the asymmetric bending mode of CF_3_/pyranose ring vibration (VI); and the asymmetric bending mode of SO_3_/pyranose ring vibration (VII). In these bands, marked differences in absorbance are observed, so the absorbance of the bands was plotted against the LiTFT salt concentration (Figure 3). It can be observed that, with the increase in the concentration of the LiTFT salt within the SBPEs, the absorbance of the band corresponding to the vibration of the OH groups (I) decreases, exhibiting a negative slope (slope = −0.074; R^2^ = 0.96), while the other bands (II to VII) show increases in absorbance. The decrease in the absorbance of the OH (I) band is attributed to the salt’s effect on the vibrations related to the oxygen atoms, specifically due to the formation of Li←O interactions, while the increase in the absorbance in bands II to VII is more related to the vibrations of the salt anions (${{CF}_{3}{SO}_{3}}^{-}$) in the SBPE matrix. 

The vibrations of bands II and V, associated with the vibrations of the functional groups of the salt, individually present positive slopes (slope = 0.189; R^2^ = 0.98 and slope = 0.135; R^2^ = 0.98, respectively) similar to the bands where the salt vibrations overlap with those of starch, as observed in bands III (slope = 0.078; R^2^ = 0.95), IV (slope = 0.074; R^2^ = 0.96), VI (slope = 0.25; R^2^ = 0.99), and VII (slope = 0.224; R^2^ = 0.98). The salt content can also affect the positions of the bands, producing slight changes in their wavenumber values, as detailed in Table 1. The differences in the positions of the bands are related to the molecular interactions between the oxygen-containing functional groups of starch and the ions (both cations and anions) produced by the dissociation of the LiTFT salt.

The differences generated in the FTIR bands due to the variations in the LiTFT salt concentrations (Figure 3) can be related to the variations in the structure of the SBPEs. The relationship between the bands assigned to the COH and COC groups, located at 1018 and 995 ${cm}^{-1}$, respectively, is crucial for understanding the structure of the starch molecules in biopolymers, as outlined in Refs. [28,36,37]. The band at 995 ${cm}^{-1}$, associated with COH vibrations, is related to the hydrogen bonds within starch, suggesting that changes in this band reflect alterations in its surrounding environment. These changes could stem from the formation of hydrogen bonds resulting from intramolecular modifications within the starch. Meanwhile, the 1018 ${cm}^{-1}$ band is related to the type-β crystallinity in starch, so it is sensitive to changes in the short-range order and, indirectly, to the proportion of amorphous starch present [28,36]. In SBPEs prepared with different concentrations of LiTFT salt, variations in the relationship between these bands were observed, indicating the effect of the salt concentration on the molecular structure within the SBPEs. The relative intensity of the COH and COC bands has been called the short-range crystallinity index [28].

**Figure 4:**
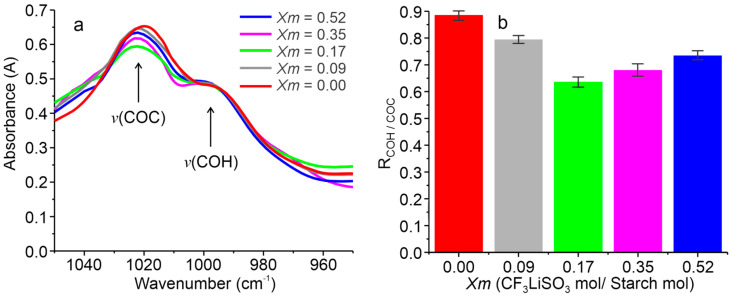
(**a**) FTIR segment from 1050 to 950 cm^−1^ of the spectra recorded in the SBPEs with different LiTFT salt concentrations and (**b**) short-range crystallinity index of the SBPEs with different LiTFT salt concentrations.

Figure 4 presents the FTIR analysis of SBPEs focusing on the region from 950 to 1050 ${cm}^{-1}$ (Figure 4a) and plots the relative intensities of the bands at 1018 and 995 ${cm}^{-1}$ ${(R}_{COH/COC})$ versus the LiTFT salt concentration in the SBPEs (Figure 4b). It can be observed that the short-range crystallinity index with the highest value was obtained in the salt-free LiTFT films (approximately 0.88). Meanwhile, the SBPEs with salt exhibited lower values, the highest being recorded at the concentration Xm = 0.09 (approximately 0.79) and the lowest at Xm = 0.17 (approximately 0.63). Notably, a marginal increase in crystallinity was detected at higher salt concentrations (Xm = 0.35 and 0.52, with approximate values of 0.68 and 0.74, respectively). These results suggest that the salt concentration can affect the structural morphology of the polymeric molecules within SBPEs. Therefore, the SBPEs with a less crystalline structure (more amorphous) are those with concentrations of Xm = 0.17, whereas those with greater crystallinity (less amorphous) are those with concentrations of Xm = 0.09. Furthermore, it is observed that, beyond a salt concentration of Xm = 0.17, the crystallinity slightly increases. Therefore, the inclusion of the LiTFT salt in SBPEs contributes to the formation of amorphous structures in starch. However, the increase in the short-range crystallinity index at concentrations of Xm = 0.35 and Xm = 0.52 suggests the generation of ion pairs that can limit the formation of amorphous structures.

### 2.2. Thermal Characterization of SBPE Films

**Figure 5:**
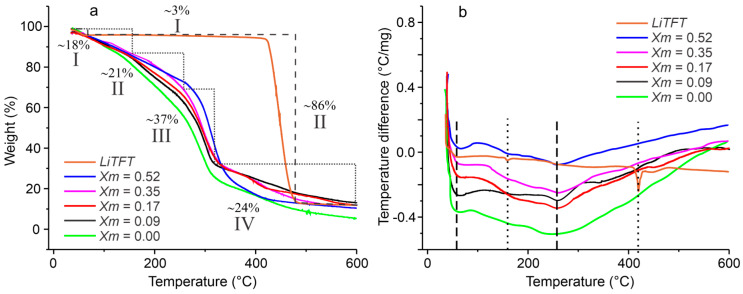
Thermal analysis of SBPEs with different LiTFT salt concentrations: (**a**) thermogravimetric analysis; (**b**) differential scanning calorimetry.

Figure 5 presents the thermograms for the TGA (Figure 5a) and DSC analyses of pristine LiTFT salt, salt-free SBPEs, and with different LiTFT salt concentrations (Figure 5b). The TGA thermograms reveal that all the films, regardless of their salt content, undergo four distinct stages of thermal degradation. The first stage of weight loss from 40 °C is caused by the loss of water (I); this process reaches 110 °C in the salt-free films and up to 115 °C in the salt-containing films, accounting for an approximate 18% weight loss. The second stage (II) is observed up to 245 °C in the salt-free films, 239 °C in the films with concentrations of Xm = 0.09, 237 °C in the films with Xm = 0.17, and 240 and 241 °C in the films with Xm = 0.35 and 0.52, respectively. This stage is attributed to the loss of the plasticizing components within the SBPEs, leading to a roughly 21% weight reduction. The third stage (III) signifies starch decomposition, spanning from the end of stage II to 317 °C for the salt-free SBPE films, 314 °C for those films with Xm concentrations of 0.09, 0.17, and 0.35, and the films with a concentration of 0.52 present a temperature of 364 °C. This process involves the degradation of the polymeric starch chains with the consequent separation of the chains and the glucose monomers that compose them. At this stage, there is a loss of approximately 37% of the weight of the films. Subsequent heating generates carbonization and ash generation in stage four (IV), where the remaining weight undergoes a further loss of around 24%.

The thermogravimetric curve of the LiTFT salt exhibits a slight weight decrease of approximately 3% from 35 to 115 °C, attributable to dehydration. This process may be related to the absorption of water from the salt during handling due to its high hygroscopicity. This result coincides with the detection of the OH band in the FTIR analysis. Additionally, a weight loss of 86% is recorded between 424 and 483 °C, corresponding to the decomposition of the salt. The thermal decomposition processes of the pristine LiTFT salt are not clearly evident in the thermogravimetric curves of the SBPEs, possibly because their mass is mainly due to starch molecules. The above may also explain that the final weight losses of the SBPEs are very similar, as is their thermogravimetric behavior. However, the presence of salt generates structural and packaging changes in the SBPEs that can manifest themselves in their thermogravimetric behavior and that are visible in the thermogravimetric curves of the biopolymers.

The differences in the temperatures of the different stages of thermal decomposition were more pronounced between the salt-free film and the films with LiTFT salt. No significant differences were observed in the decomposition temperatures among the films with varying salt concentrations. This phenomenon may be attributed to the more marked difference in the molecular packing between the salt-free films and those with some salt content. The greatest differences are evident in stages II and III, with the highest salt concentration (Xm = 0.52) showing the most pronounced differences. In general, the differences in the molecular packing in the films with distinct salt concentrations do not lead to substantial changes (≤5 °C) in their degradation temperatures.

In the thermograms recorded by DSC (Figure 5b), two endothermic processes are evident. The first process, attributable to the breakdown of the crystallinity and packing of the starch during retrogradation, occurs at 70.1 °C in the SBPE films that are salt-free and at 68.1, 67.9, 67.7, and 67.4 °C in the films with concentrations of Xm = 0.09, 0.17, 0.35, and 0.52, respectively. This represents a decrease of between 2 and 3 °C for the SBPEs with salt in relation to the salt-free SBPEs and a variation of 0.2 °C between the films with different salt concentrations. The second endothermic process is generated by the breaking of the starch chains into their glucose units and occurs consistently at 271.5 ± 0.6 °C across all the SBPEs. The temperature differences in the first process may be attributed to the differences in the ordered and amorphous structures caused by the amount of salt in the films as this first process is directly related to the breakdown of the crystalline order of the SBPEs. The second process seems not to be affected by the presence of salt or its concentration, perhaps because this endothermic process is unrelated to the morphology of the SBPEs—where the salt content has its greatest effect—but rather related to the fragmentation of the polymer chains.

### 2.3. Voltammetric Characterization of Cassava Starch SBPE Films

**Figure 6:**
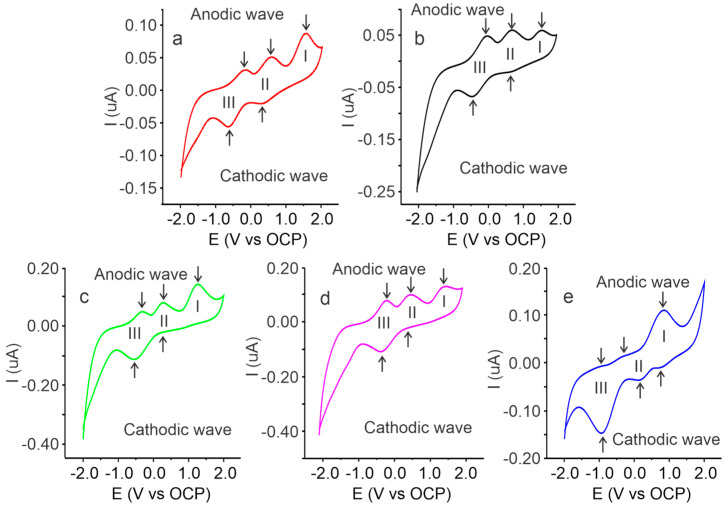
Cyclic voltammetry signals of the SBPEs with different LiTFT salt concentrations: (**a**) Xm = 0.00, (**b**) Xm = 0.09, (**c**) Xm = 0.17, (**d**) Xm = 0.35, and (**e**) Xm = 0.52. Roman numbers indicate the redox processes.

The voltammetric responses of the SBPEs with different LiTFT salt concentrations are presented in Figure 6. It can be seen that all the SBPEs, independent of the amount of salt, showed redox activity with the oxidation processes in the anodic wave and reductions in the cathodic wave. The voltammetry of the cassava starch salt-free SBPE film shows three oxidation peaks in the anodic wave at 1.52, 0.56, and −0.19 V for processes I, II, and III, respectively, and, in the cathodic wave, the peak reduction in process II to 0.31 V and process III to −0.66 V.

The voltammogram of the SBPE with a salt concentration of Xm = 0.09 exhibits three anodic peaks at 1.54, 0.70, and −0.02 V in processes I, II, and III, respectively, along with two cathodic peaks at 0.64 and −0.41 V in processes II and III. The SBPE with the LiTFT at a concentration of Xm = 0.17 shows an oxidation peak at 1.26 V in process I, a redox pair in II with an anodic peak at 0.26 V and a cathodic peak at 0.20 V, and, in process III, an anodic peak at −0.32 V and cathodic peak at −0.55 V. The signal for the SBPE with a salt concentration of Xm = 0.35 reveals three oxidation peaks in the anodic wave at 1.50, 0.54, and 0.13 V (processes I, II, and III) and two cathodic peaks at 0.43 and −0.26 V (processes II and III). The voltammetric response of the SBPE film with Xm = 0.52 shows three anodic and cathodic peaks; in the redox pair of process I, an anodic peak at 0.85 V and a cathodic peak at 0.80 V are observed, process II presents its anodic and cathodic potentials at −0.21 and 0.12 V, and process III at 0.85 and −0.90 V, respectively.

The redox processes observed in the voltammograms are due to the oxidation and reduction reactions of the glucose units of the starch. It has been reported that glucose can undergo three redox processes [34,38]. The mechanism of these reactions includes a dehydrogenation reaction of glucose at its C1 carbon, observed in process III; a redox reaction of glucose facilitated by hydroxyl ions produced from the dissociation of water molecules, occurring in process II; and a reaction of glucose with the metal oxides present on the surface of the electrodes (process I). The peaks of the oxidation and reduction processes of the films with different LiTFT salt concentrations show different potentials, suggesting that the amount of salt used affects the redox properties and their potentials. The potential and current values of the anodic and cathodic peaks of the voltammetric signals for the SBPEs with different LiTFT salt concentrations are summarized in Table 2.

The peak currents (Ip) present variations in their intensity values when the salt concentration in the SBPEs changes. This parameter is related to the faradaic current, which for any electrochemical reaction is proportional to the electrode area, the concentration or amount of redox species (oxidizable or reducible), and the number of electrons transferred in the redox reactions or processes. Given that the chemical species involved in the redox reactions is glucose and the distinguishing factor in the SBPEs studied is the LiTFT salt concentration, the differences in the Ip values are primarily related to the availability of glucose molecules to be oxidized or reduced on the surface of the electrode (i.e., the amount of redox species) as the other parameters (area of the electrodes and number of electrons transferred in the redox reactions) remain constant. The above may indicate that differences in the salt concentrations in SBPEs can affect the availability of glucose molecules, which is related to the amorphous and/or crystalline structure of the starch chains. These structural differences can either enable or limit the access to glucose units, thereby generating variations in the potential (Ep) and peak current (Ip) values. It can be seen in Table 2 that, at higher concentrations of LiTFT salt, lower oxidation and reduction potentials (Epa and Epc) are presented, which indicates that glucose redox reactions are facilitated by a greater amount of salt. The above can be favorable in applications of these materials in electrochemical sensors. Additionally, it can be seen that the currents of the oxidation and reduction peaks (Ipa and Ipc) present an asymmetric trend. It increases up to the concentration of Xm = 0.17 and then decreases at higher concentrations. This behavior may be due to the effect of the salt concentration on the morphology since, as previously discussed in the FTIR analysis, the amorphous structures are greater at this concentration, which facilitates the movement of the ions and favors the flow of the current.

### 2.4. Electrochemical Impedance Spectroscopy Characterization of SBPE Films

**Figure 7:**
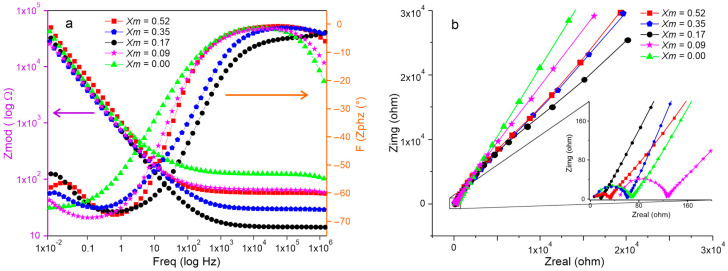
(**a**) Bode plots and (**b**) Nyquist plots of SBPE films with different LiTFT concentrations.

Electrochemical impedance spectroscopy was used to evaluate the effect of the salt concentration in SBPE films on their electrochemical behavior. Figure 7a shows the Bode plot, which represents the impedance modulus (Zmod) on a logarithmic scale on the Y1 axis (first ordinate) and the phase change (Zphz) on the Y2 axis (second ordinate) versus the logarithm of the frequency on the abscissa axis (X). It can be seen that, regardless of the salt amount, all the films exhibit similar curve behaviors; at low frequencies, the impedance modulus is notably greater than at high frequencies. At low frequencies, the impedance modulus values were close to 0.1 MΩ, decreasing to between 0.1 and 0.01 kΩ at high frequencies. Concurrently, the phase change (Zphz) presented values close to 0° at high frequencies, whereas, at low frequencies, the values range between −55 and −65°. This behavior shows that, at low frequencies, the SBPE films are more capacitive, characterized by a negative impedance phase change typical of capacitive behaviors. Meanwhile, at high frequencies, the impedance phase change approaches 0°, evidencing the current flow through the films and resistive behavior. In Figure 7b, the Nyquist plot of the SBPE samples is presented. It can be observed that the spectrum curves have similar trends; a depressed semicircle is appreciated when enlarging the high-frequency area and continues with a tail or growing line at low frequencies, which in all cases describe angles slightly greater than 45°. The formation of the semicircle is attributed to the combination of the capacitive and resistive processes or elements in the SBPEs. Meanwhile, the growing line reflects the ionic diffusion processes.

**Figure 8:**
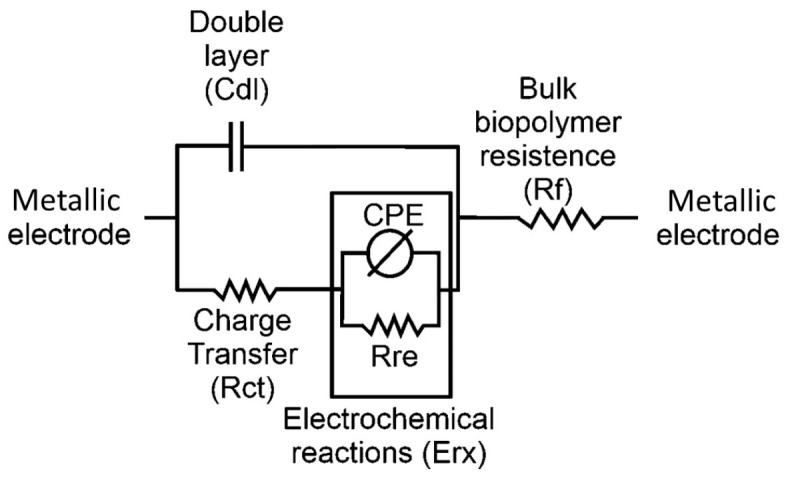
Equivalent circuit model of SBPEs with different LiTFT concentrations.

From the impedancemetric behavior, the equivalent circuit of the electrochemical system was established. Figure 8 shows the equivalent circuit estimated from the impedance spectra recorded for the SBPEs with different LiTFT salt concentrations, represented as/Rf-(Cdl/(Rct-(CPE/Rre))). The circuit comprises a capacitive element (Cdl), arranged in parallel with a resistance (Rct), which are attributed to the phenomena of double-layer formation and electron transfer occurring at the electrode/biopolymer interface. Additionally, there is a parallel system composed of a constant phase element (CPE) and a resistance (Rre). This configuration is related to the abnormal transport of electrons toward the biopolymeric matrix, a behavior that is generated by the oxidation/reduction processes of glucose units, as evidenced in cyclic voltammetry. The resistance generated by the movement of the charges through the biopolymeric matrix is represented in the equivalent circuit by the bulk resistance of the films (Rf). The values of the electrochemical parameters calculated from the equivalent circuit are presented in Table 3.

The capacitance of the double layer in SBPE films registers higher values with increasing salt concentrations, suggesting that this may be due to an increase in the number of ions at the electrode/biopolymer interface. Correspondingly, the resistance to electron transfer at this interface is higher in the SBPEs containing salt, with the highest resistance observed at a salt concentration of Xm = 0.17 and the lowest in the films synthesized without salt (Xm = 0.0).

Additionally, the CPE/Rre system related to the redox processes in the films also shows differences due to variations in the LiTFT salt content. This redox activity was evidenced in the voltammetric analysis and has been previously reported in SBPEs elaborated from starch [18,39]. The CPE exhibits a trend parallel to the double-layer capacitance, showing a progressive increase with rising salt concentrations. Meanwhile, the resistance Rre increases with the addition of salt, with a maximum value at Xm = 0.17, which decreases at concentrations higher than Xm = 0.35 and 0.52. The bulk resistance (Rf), related to the electrical resistivity of the films, shows higher resistance values in the salt-free SBPEs. Conversely, in the SBPEs containing salt, the resistance is lower, with the lowest resistance observed in films at a concentration of Xm = 0.17, increasing again at higher salt concentrations.

The bulk resistance (Rf) of the films is used to calculate the ionic conductivity of the SBPEs using the equation σ=l/Rf.A, where *l* is the thickness of the film, *Rf* the bulk resistance, and *A* the contact area between the films and the electrodes. The ionic conductivity of the salt-free SBPE film was measured at 5.80 × 10^−4^ S cm^−1^, while the films with added salt showed a significant increase (by one magnitude order), with values of 1.38 × 10^−3^, 2.72 × 10^−3^, 2.10 × 10^−3^, and 1.89 × 10^−3^ S cm^−1^, for the films with concentrations of Xm = 0.09, 0.17, 0.35, and 0.52, respectively. The ionic conductivity observed in the salt-free SBPE films can be attributed to the movement of the H+ charges generated by the dissociation of the OH groups in the biopolymer matrix. It has been reported that starch, when in contact with water, behaves as a weak polyacid since it has three OH (hydroxyl) groups per glucose unit. Starch has a PKa of 13 at 40 °C, so the hydroxyl groups (OH) are completely ionized during its manufacturing process, where it is heated to 75 ± 5 °C, generating H+ ions. The above generates free charges in the matrix that can enable the ionic conductivity of the salt-free SBPE films. The conductivities of the SBPEs doped with LiTFT with different concentrations are higher than those reported by other authors for starch SBPEs. For example, the SBPEs from starch–LiPF_6_, starch/Ch–KI, and starch/Ch–NH_4_I–gly achieved conductivities of 1.47 × 10^−4^, 4.65 × 10^−4^, and 1.28 × 10^−3^ S cm^−1^, respectively [40,41,42].

The highest conductivity of 2.72 × 10^−3^ S cm^−1^ is observed in films with a molar ratio of 0.17; however, it decreases in the films with a greater amount of salt with molar ratios of 0.35 and 0.52. This trend can be explained by the influence of the amorphous structure on the charge mobility, which enhances the conductivity by providing more free spaces for ion movement. The addition of salts favors the amorphous nature of SBPEs, facilitating ion movement and improving the ionic conductivity within the biopolymeric matrix. However, an excessive salt concentration can lead to the formation of ionic pairs, which may impede the ion movement through the biopolymer, resulting in a decrease in the ionic conductivity. This decrease in conductivity at higher salt concentrations (Xm = 0.35 and 0.52) is consistent with the formation of ionic aggregates within the SBPEs due to the increased LiTFT concentration, reflecting the short-range crystallinity index previously discussed. This helps to explain the observed reduction in conductivity at these higher concentrations.

## 3. Materials and Methods

### 3.1. Reagents and Materials

All reagents used were of analytical grade and purchased from Merck (Darmstadt, Germany); glycerol (99.5%), glutaraldehyde (70.0%), and lithium trifluoromethanesulfonate/i.e., lithium triflate (99.9%). The processes employed ultrapure water, filtered using a Stakpure OmniaTap 6 purification system (Niederahr, Germany). The cassava starch used in the production of the biopolymers was obtained in the laboratory.

To extract cassava starch, 500 g of cassava tuber of the Manihot esculenta Crantz variety were weighed, washed, peeled, and disintegrated in an industrial processor at 25,000 rpm with 1 L of water. This mixture was then filtered through a sieve (0.2 mm) three times until no visible starch residue remained in the water. The aqueous starch mixture was allowed to decant, the supernatant was discarded, and the collected starch was washed thrice with water. The washed starch was dried in an oven at 40 °C and finally sieved (0.05 mm) to produce a bright white powder. The starch’s purity was determined to be 99.3%, verified by the standard methods of the International Association of Analytical Chemists.

### 3.2. Preparation of Cassava Starch SBPE Films with Different LiTFT Salt Concentrations

Cassava starch SBPE samples were prepared in films format utilizing a thermochemical procedure. Initially, 3 g of cassava starch were mixed in 100 mL of water with stirring at 1500 rpm. This mixture was heated to 75 ± 5 °C for 15 min with constant stirring to induce gelatinization, after which it was allowed to cool to ambient temperature. Then, 1.5 g of glycerol, 1.5 g of polyethylene glycol, 1.0 g of glutaraldehyde, and varying concentrations of the lithium triflate salt (lithium trifluoromethanesulfonate) were added. The samples were prepared with different concentrations (Xm = moles of salt/moles of starch) of lithium salt, including 0.0 (sample salt-free), 0.09, 0.17, 0.35, and 0.52. The mixtures were again stirred at 1500 rpm and reheated at 75 ± 5 °C for 15 min to obtain a completely homogeneous solution. Once cooled to room temperature, the mixtures were poured into Teflon Petri dishes and left in an oven at 70 °C for 48 h to evaporate any remaining solvent. After the drying procedure, the samples were cooled to room temperature and conditioned for 24 h before being subjected to various analyses. This process was replicated five times for each sample concentration.

### 3.3. Characterization of Cassava Starch SBPEs

#### 3.3.1. Fourier Transform Infrared (FTIR) Spectroscopy

Infrared spectroscopy was conducted using a Perkin-Elmer Spectrum Two infrared spectrometer equipped with an ATR (attenuated total reflectance) device (Shelton, CT, USA). The spectra were recorded at room temperature, covering a wavenumber range from 4000 to 400 ${cm}^{-1}$, with a mirror speed set at 0.4 cm/s and a resolution of 4 ${cm}^{-1}$. To ensure accuracy, the FTIR spectra of the samples were obtained after recording the background of the equipment.

#### 3.3.2. Thermal Analysis

The thermal analysis was performed by thermogravimetric analysis (TGA) and differential scanning calorimetry (DSC) simultaneously, using a Perkin-Elmer STA 6000 thermal analyzer (Shelton, CT, USA). Before analysis, the equipment was calibrated using a standard indium sample to ensure precision. The samples of SBPEs were analyzed without any prior treatment to assess their thermal properties directly. For the analysis, sample masses ranging from 1.5 to 2.0 mg were placed in a ceramic pan. The experiments were carried out with a heating rate of 10 °C/min, under a high purity nitrogen atmosphere (99.9%) at a flow rate of 20 mL/min, across a temperature spectrum of 40 to 600 °C.

#### 3.3.3. Cyclic Voltammetry

The redox behavior of the SBPEs was studied by cyclic voltammetry. Cyclic voltammetry was carried out with a Gamry 1100E potentiostat/galvanostat (Warminster, PA, USA) controlled with the Gamry instruments framework V 7.10 software (Version 7.10). The measurements were carried out using a dry measurement cell, taking 1 cm^2^ samples and sandwiching between two stainless steel sheets. A potential range of −2 to 2 V was used, with a scan rate of 10 mV/s, and the open circuit potential (0.10 V) was used as the reference potential. The voltammetric curves were analyzed using the Echem Analyst software (version 7.8) to determine the potentials and currents of the redox peaks.

#### 3.3.4. Electrochemical Impedance Spectroscopy

Electrochemical impedance spectroscopy (EIS) was carried out using a Gamry 1100E potentiostat/galvanostat (Warminster, PA, USA) controlled with the Gamry instruments framework V 7.10 software (Version 7.10). The measurements were carried out in a frequency range of 10 mHz to 2 MHz and an AC voltage of 10 mV rms. The samples were cut into 1.0 cm^2^ squares and sandwiched between two stainless steel plates. The impedance spectra were analyzed with the Echem Analyst software (version 7.8) to determine the equivalent circuit model and the determination of the electrochemical parameters.

## 4. Conclusions

The SBPEs elaborated with different LiTFT salt concentrations displayed distinct features in their FTIR spectra, particularly the bands corresponding to both starch and LiTFT salt. The amount of salt incorporated affects the intensity of the starch bands related to oxygen atoms, generating variations in their absorbances and, in some cases, changes in the wavenumber. Additionally, the FTIR spectra further facilitated the determination of the short-range crystallinity index, through the ratio of the 995 to 1020 cm^−1^ band, indicating that the salt content significantly affects the morphology of the polymer chains by promoting the formation of amorphous structures.

The addition of salt into the films results in a slight decrease in the thermal stability (approximately 5 °C). However, the amount of salt added does not markedly affect their thermal stability. The voltammetric behavior of the SBPEs, influenced by the varying salt concentrations, showed differences in the peak potentials (Ep) and peak currents (Ip). These differences are primarily attributed to the structural variances in the crystalline morphology, which either facilitate or hinder access to the glucose units undergoing redox processes. The effect of the amount of salt on the morphology also influenced the electrochemical parameters determined through impedance studies. Impedance spectroscopy enabled the establishment of an equivalent circuit model Rf–(Cdl/(Rct–(CPE/Rre))). This model facilitated the determination of the electrochemical parameters, revealing enhanced conductivity in the SBPEs with Xm = 0.17. The salt concentrations exceeding this value exhibited lower conductivity due to the recrystallization of excess salt in the biopolymeric matrix.

It was possible to establish that the addition of salt increases the ionic conductivity of the SBPEs and that it favors electroactivity, which may enable the application of these materials in the development of electrochemical sensors. Furthermore, it was shown that the LiTFT salt concentration affects the ionic conduction, thermal stability, electroactivity, and the structure of the cassava starch SBPEs, which enables us to establish the bases for the modularization and optimization of the properties of these materials when applied in the development of technological devices that require specific performance characteristics.

## Figures and Tables

**Table 1:** Vibration bands of the spectra of pure LiTFT, and salt-free SBPEs and with salt at different concentrations.

Assignments	$\mathrm{Wavenumber}\;({cm}^{-1}$) of SBPEs Xm = [LiTFT mol/starch mol]					LiTFT
	0.00	0.09	0.17	0.35	0.52	
O–H stretching	3364	3371	3377	3385	3399	3486
C–H stretching	2926–2878	2927–2878	2927–2878	2927–2878	2927–2878	-
C=O stretching vibration	1713	1713	1713	1713	1713	-
O–H (water) bending/O-H bending (sulfonic)	1649	1648	1648	1648	1648	1647
C–H bending	1456	1456	1456	1456	1456	
O–H bending	1407	1407	1408	1408	1408	
COH bending/S=O stretching	1350	1350	1350	1350	1350	
CH_2_OH related modes/Asymmetric stretching mode of SO_3_	1247	1279–1250	1279–1250	1279–1250	1279–1250	1292–1248
COH deformation/Symmetric stretching mode CF_3_	1202	1219	1219	1219	1219	1229
Asymmetric stretching mode of CF_3_	-	-	-	-	-	1182
CO antisymmetric bridge stretching	1146	1145	1143	1142	1142	-
COH antisymmetric stretching in plane ring	1103	1103	1102	1102	1103	-
C–OH bending	1077	1078	1077	1077	1076	-
Symmetric stretching mode of SO_3_	-	-	-	-	-	1039
COC ring vibration of Carbohydrate	1018	1021	1021	1021	1021	-
COH solved	995	998	998	998	998	-
C-H bending modes	844	843	843	843	843	-
Symmetric bending mode CF_3_	-	-	-	-	-	773
CH_2_ rocking	757	757	757	757	757	
Symmetric bending mode SO_3_		639	639	638	638	627
Asymmetric bending mode CF_3_/pyranose ring vibration	570	571	571	571	571	574
Pyranose ring vibration	522	520	517	516	515	
Asymmetric bending mode SO_3_	-	-	-	-	-	512

**Table 2:** Peak potential (Ep) and peak current (Ip) values of the oxidation/reduction processes of SBPEs with different LiTFT salt concentrations.

Process	Peaks		Cassava Starch SBPEs with Different Salt Concentrations (Lithium Triflate)				
			Xm = 0.00	Xm = 0.09	Xm = 0.17	Xm = 0.35	Xm = 0.52
**I**	Anodic	Ep (V)	1.52	1.54	1.26	1.50	0.85
		Ip (µA)	0.096	0.067	0.144	0.132	0.111
	Cathodic	Ep (V)	-	-	-	-	0.80
		Ip (µA)	-	-	-	-	−0.010
**II**	Anodic	Ep (V)	0.56	0.70	0.26	0.54	−0.21
		Ip (µA)	0.055	0.069	0.083	0.104	0.014
	Cathodic	Ep (V)	0.31	0.64	0.20	0.43	0.12
		Ip (µA)	−0.028	−0.012	−0.022	−0.019	−0.038
**III**	Anodic	Ep (V)	−0.19	−0.02	−0.32	0.13	−0.85
		Ip (µA)	0.032	0.058	0.052	0.087	−0.005
	Cathodic	Ep (V)	−0.66	−0.41	−0.55	−0.26	−0.90
		Ip (µA)	−0.070	−0.059	−0.112	−0.108	−0.014

**Table 3:** Values of electrochemical parameters obtained from the impedance spectroscopy signals of the SBPEs with different LiTFT salt concentrations.

Salt Concentration (Xm)	Electrochemical Parameters				
	Cdl (F)	Rct (Ω)	Rre (Ω)	CPE (S*s^a^)	Rf (Ω)
0.00	2.27 × 10^−4^	5.19 × 10^2^	1.26 × 10^5^	2.10 × 10^−6^	8.96 × 10^2^
0.09	2.37 × 10^−4^	6.70 × 10^4^	7.73 × 10^5^	1.17 × 10^−4^	3.75 × 10^2^
0.17	2.75 × 10^−4^	3.10 × 10^7^	1.01 × 10^7^	1.67 × 10^−2^	1.91 × 10^2^
0.35	3.38 × 10^−4^	1.89 × 10^6^	1.41 × 10^4^	6.13 × 10^−2^	2.48 × 10^2^
0.52	3.54 × 10^−4^	1.16 × 10^6^	1.12 × 10^4^	5.00 × 10^−1^	2.75 × 10^2^

## Data Availability

Dataset available on request from the authors.
